# Subchondral osteoclasts and osteoarthritis: new insights and potential therapeutic avenues: The role of OCs in OA

**DOI:** 10.3724/abbs.2024017

**Published:** 2024-03-05

**Authors:** Wenlong Chen, Qiufei Wang, Huaqiang Tao, Lingfeng Lu, Jing Zhou, Qiang Wang, Wei Huang, Xing Yang

**Affiliations:** 1 Orthopedics and Sports Medicine Center Suzhou Municipal Hospital Nanjing Medical University Affiliated Suzhou Hospital Suzhou 215000 China; 2 Gusu School Nanjing Medical University Suzhou 215000 China; 3 Department of Orthopedics the First Affiliated Hospital of Soochow University Suzhou 215000 China; 4 Department of Orthopaedics the First Affiliated Hospital of USTC Division of Life Sciences and Medicine University of Science and Technology of China Hefei 230026 China

**Keywords:** osteoarthritis, osteoclast, subchondral bone

## Abstract

Osteoarthritis (OA) is the most common joint disease, and good therapeutic results are often difficult to obtain due to its complex pathogenesis and diverse causative factors. After decades of research and exploration of OA, it has been progressively found that subchondral bone is essential for its pathogenesis, and pathological changes in subchondral bone can be observed even before cartilage lesions develop. Osteoclasts, the main cells regulating bone resorption, play a crucial role in the pathogenesis of subchondral bone. Subchondral osteoclasts regulate the homeostasis of subchondral bone through the secretion of degradative enzymes, immunomodulation, and cell signaling pathways. In OA, osteoclasts are overactivated by autophagy, ncRNAs, and Rankl/Rank/OPG signaling pathways. Excessive bone resorption disrupts the balance of bone remodeling, leading to increased subchondral bone loss, decreased bone mineral density and consequent structural damage to articular cartilage and joint pain. With increased understanding of bone biology and targeted therapies, researchers have found that the activity and function of subchondral osteoclasts are affected by multiple pathways. In this review, we summarize the roles and mechanisms of subchondral osteoclasts in OA, enumerate the latest advances in subchondral osteoclast-targeted therapy for OA, and look forward to the future trends of subchondral osteoclast-targeted therapies in clinical applications to fill the gaps in the current knowledge of OA treatment and to develop new therapeutic strategies.

## Introduction

Osteoarthritis (OA) is one of the most common causes of disability among elderly individuals worldwide. It is essentially a degenerative disease involving lesions of the cartilage, subchondral bone, synovium, meniscus, and surrounding tissues [1]. Currently, the incidence of OA is increasing gradually, and most OA patients are elderly. It is estimated that one-third of people over the age of 65 suffer from OA, with a higher incidence among women than men [2]. The causes of OA are complex and varied and are influenced by a variety of factors, such as sex, age, genetics, diet and obesity; among them, population aging and obesity are the most important factors. Moreover, abnormal mechanical cues, such as joint instability, overuse of joints, and imbalance of muscle strength, are also important factors in the development of OA, and sports injuries can lead to structural and weight-bearing abnormalities in joints, increase the risk of cartilage damage, and contribute to OA [3]. Osteophytes are a prevalent anatomical manifestation of OA. They are believed to originate from cells in the periosteum. The process of osteophyte formation shares similarities with the process of bone repair observed during fracture healing, where the periosteum is deemed crucial. Stem cells derived from the periosteum, known as periosteum-derived cells (PDCs), reside in the cambium periosteum layer and exhibit pluripotent characteristics. PDCs play a pivotal role in bone repair and can be mobilized in response to inflammatory reactions and mechanical stimuli. Abnormal mechanical stress not only induces OA but also accelerates and promotes the production of bone encumbrances, which exacerbates the pain of OA patients [ 4, 5].

Joint pain and loss of function are the main reasons for treating OA. Common treatments include nonpharmacological treatments, such as weight control and physiotherapy. Pharmacological treatments include nonsteroidal anti-inflammatory drugs (NSAIDs), glucocorticoids, and hyaluronic acid, whereas surgical treatments include joint replacement. Due to the numerous pathogenic factors and complex pathological processes involved, conventional treatments can alleviate only the symptoms of OA but cannot reverse the progression of OA or restore normal joint structure [ 6– 8]. Traditional therapies are frequently accompanied by serious effects. NSAIDs can relieve pain and inflammation, but long-term use may lead to gastrointestinal issues such as gastric ulcers and bleeding, and long-term use of anti-inflammatory drugs may also increase cardiovascular risk. Physiotherapy may initially cause some discomfort and pain but is generally safe; excessive exercise or improper management may aggravate joint damage. Surgical treatment may be accompanied by certain surgical risks, such as infection, bleeding, and anesthesia reactions; in addition, there are various problems, such as loosening of the artificial joint, joint durability, difficulty in recovery, and lack of function [ 9, 10]. Therefore, new treatment methods are urgently needed.

Osteoclasts are indispensable in many bone-related diseases, including osteoporosis, osteosclerosis, bone tumors, rheumatoid arthritis (RA), and Paget’s disease. These diseases usually involve abnormalities in bone structure, density, metabolism, or function [11]. In osteoporosis, overactivated osteoclasts absorb excess calcium and phosphorus from bone tissue, leading to decreased bone mineral density and fragility [ 12, 13]. Osteosclerosis, a genetic disorder characterized by increased bone mass, is caused by defects in osteoclast formation and function [ 14, 15]. In addition, Scr kinase deficiency has been reported to affect osteoclast activity, leading to osteoporosis [16]. Scr kinase deficiency leads to a lack of intact folds in osteoclasts, which impacts the adequate contact of osteoclasts with the bone surface and their ability to absorb minerals and proteins from bone tissue [ 17, 18]. In the case of bone tumors, surrounding malignant cells may activate osteoclasts, leading to destruction of the bone structure and irritation of nerve endings. Patients with bone tumors may experience bone-related pain [19]. Additionally, there are interactions between osteoclasts and malignant bone tumor cells. Tumor cells may produce chemokines that attract osteoclasts to migrate around tumors, thereby accelerating the destruction of bone tissue around tumors and enhancing the invasive ability of bone tumors [ 20, 21]. The primary function of osteoclasts in RA is related mainly to joint destruction and pain. During RA, inflammatory cells stimulate osteoclasts to release enzymes and cytokines related to bone resorption, destroy bone tissues, alter the joint microenvironment, and aggravate inflammation, resulting in severe joint damage and pain [ 22– 25]. The activity and number of osteoclasts in Paget’s disease are markedly increased, which leads to changes in bone mineral density and greatly increases the risk of fracture; concurrently, altered bone mineral density greatly increases the risk of fracture due to the significantly increased activity and number of osteoclasts. Moreover, excessive bone resorption increases the production rate of new bone tissue, but the new bone is usually arranged unevenly, resulting in an irregular bone shape and abnormal bone remodeling, which can cause bone deformity and pain [ 26– 28].

A growing body of evidence indicates that osteoclasts play an important role in the development of many diseases. However, the functions and mechanisms of action of subchondral osteoclasts in OA remain unclear. Therefore, this review summarizes the roles and mechanisms of subchondral osteoclasts in OA progression and regulation to provide new targets for treating OA.

## Composition of Subchondral Bone

**Figure 1:**
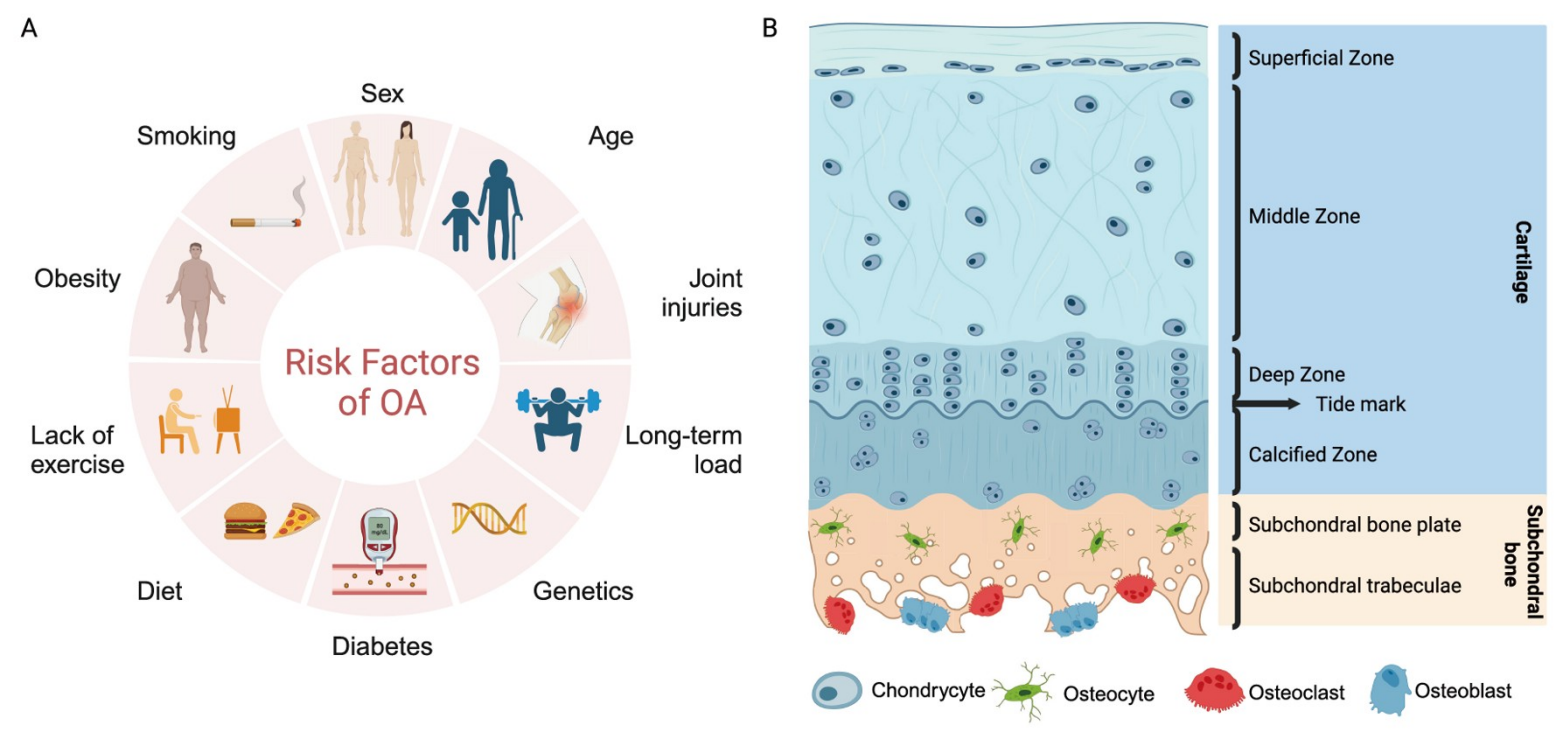
Figure 1 Many pathological factors, such as smoking, obesity, and aging, are involved in the progression of OA These unfavorable factors mediate numerous pathological molecular signals within the knee joint, causing imbalances in multiple cellular homeostasis pathways (osteoclast, osteocyte, osteoblast, etc.) in the bone microenvironment and further exacerbating disease progression.

The subchondral bone is located distal to the calcified cartilage. It usually plays a role in maintaining joint elasticity, supporting articular cartilage, and influencing cartilage metabolism [29]. It can generally be divided into two anatomical entities: the subchondral bone plate and subchondral trabecular bone [30] ( Figure 1). The subchondral bone plate consists of a thin layer of cortical bone adjacent to calcified cartilage. The cortical bone plate has distinct pores and is a permeable structure that provides a direct connecting channel between the articular cartilage and subchondral trabeculae, through which many blood vessels and nerves pass. In contrast, the subchondral trabeculae are composed of cancellous bone close to the bone marrow cavity; this bone marrow has a sparser structure, is metabolically active, and is rich in blood vessels and nerves [ 31, 32]. Subchondral bone contains a variety of cells with different functions, including osteoclasts, osteocytes, osteoblasts, and endothelial cells. They collectively influence the microstructure and histopathological changes in subchondral bone through cell-mediated remodeling and modeling processes, and the four types of cells in subchondral bone interfere with each other [ 33, 34]. These cells are described below:

### Osteoclast

Osteoclasts are specialized cells with multiple functions that are primarily responsible for bone resorption and remodeling to maintain the normal physiological state of the skeleton. They control bone growth and renewal by binding to the bone surface and releasing acids and enzymes from lysosomes to degrade and dissolve inorganic salts and organic matrices in the bone tissue [ 35, 36]. Osteoclasts are closely associated with the immune system and secrete cytokines and growth factors in response to proinflammatory stimuli [37]. Moreover, osteoclasts are essential for bone metabolism and angiogenesis. On the one hand, osteoclasts control the endocrine regulation of calcium and phosphate by regulating their response to parathyroid hormone and calcitonin; on the other hand, osteoclasts promote angiogenesis by facilitating endothelial cell migration and stimulating the paracrine secretion of endothelial cells to increase vascular endothelial growth factor (VEGF) [ 38, 39]. In addition, osteoclasts regulate osteoblast maturation and differentiation through RANK/RANKL/OPG, Ephrinb2-Ephb4 signaling, sphingolipid signaling, and other membrane-associated proteins [40]. Osteoclasts play an important role in many diseases. However, the function of subchondral osteoclasts in OA remains unclear.

### Osteocyte

Osteocytes are the most abundant cells in bone and account for 90%–95% of bone cells. Osteocytes are responsible for maintaining bone homeostasis and mechanotransduction. They are the primary regulators of osteoblasts and osteoclasts. Upon stimulation, osteocytes maintain bone homeostasis by regulating the signals generated by mechanical loads and recruiting osteoclasts and osteoblasts to initiate the repair process [ 41, 42]. Osteocytes also modulate the extracellular matrix through specific molecular remodeling mechanisms. As endocrine cells, osteocytes can further influence bone metabolism by affecting phosphate uptake, insulin secretion, and skeletal muscle function, thereby regulating bone size and shape. Previous studies have demonstrated that osteocytes are crucial for bone aging [ 43, 44]. In addition to these functions, many other functions of osteocytes, such as interactions with the immune system [45], influencing hematopoiesis through the secretion of cytokines [46], and promoting the progression of bone cancer, are still being investigated [47].

### Osteoblast

Osteoblasts are derived from bone marrow mesenchymal stem cells (BMSCs), and their differentiation is a key step in osteogenesis. There are three stages of differentiation from BMSCs to osteoblasts: osteogenitor cells, preosteoblasts, and osteoclasts [ 48, 49]. This differentiation process is regulated by various transcription factors, signaling pathways, and genes, such as bone morphogenetic proteins (Bmp), Runx2 transcription factors, and the Wnt signaling pathway [ 50, 51]. Osteoblasts play an important role in bone development and the maintenance of homeostasis. VEGF-derived proteins affect bone repair and regeneration and contribute to bone defect healing by stimulating vascular and osteoclast recruitment [52]. Osteoblasts, which have abundant basophilic cytoplasm, a large number of mitochondria, and high Golgi capacity, produce a unique extracellular protein assemblage consisting of large amounts of collagen type I, osteocalcin, alkaline phosphatase, and the extracellular matrix [53] and simultaneously affect the development and differentiation of osteoclasts. Previous studies have demonstrated that osteoblasts can influence the cellular behavior, survival, and differentiation of osteoclasts through direct contact between osteoblasts and osteoclasts through the bidirectional transactivation of activation signals such as EFNB2-EPHB4 and FASL-FAS or secreted proteins such as PANKL/OPG, M-CSF, Wnt5a, and Wnt16 [ 54, 55].

### Endothelial cell

Subchondral bone endothelial cells are smooth monolayers of cells tightly arranged in the lining of the vascular lumen that serve various important physiological functions. Endothelial cells can regulate angiogenesis and blood flow by secreting vascular and secretory factors or engaging in molecular crosstalk with osteoblasts, playing a crucial role in fracture healing and the maintenance of bone homeostasis [ 56, 57]. Endothelial cells can participate in the inflammatory response in the subchondral bone region in various ways, such as through the calcium signaling pathway, which produces several immune factors and chemokines that direct immune cells to the damaged site and promote tissue repair and regeneration [58]. Simultaneously, endothelial cells produce a variety of cytokines, such as basic fibroblast growth factor (bFGF) and ADAMTS, thereby promoting the proliferation and differentiation of BMSCs and maintaining the regenerative capacity of bone tissue [ 59, 60]. Additionally, endothelial cells partially regulate the maturation and differentiation of osteoblasts. Endothelial cells can produce a variety of cytokines and growth factors, such as VEGF, or through cell crosstalk to influence osteoblasts [61].

## Origin of Osteoclasts

Since the end of the 19th century, when osteoclasts were first discovered and observed under a microscope, the multinucleated morphology of osteoclasts has given rise to a great deal of discussion about their origin and function. Since then, various experiments have been conducted, and many theories have been proposed to explore and explain the origin of osteoclasts. As common osteocytes and osteoblasts are involved in the regulation of bone remodeling, it was initially thought that there was a commonality between the two in the early 20th century. However, a growing body of evidence is beginning to support a ‘biphyletic origin’ theory between the two types of osteocytes, and morphological similarities have been observed between mature osteoclasts and macrophage-derived cells [62].

The hematopoietic origin of osteoclasts was confirmed by Walker’s pioneering experiments in the 1970s, in which cells from the spleen and bone marrow of normal mice were transplanted into mice suffering from hereditary osteosclerosis and osteoclast deficiency. As a result, bone resorption was restored in the mice, suggesting that the hematopoietic organs could produce certain cells to resorb hardened bone tissue [63]. Subsequently, an increasing number of scholars have shown that osteoclast production is inextricably linked to monocyte/macrophage production. Scheven *et al*. [64] demonstrated that populations of hematopoietic stem cells (HSCs) can produce osteoclasts and that the ability to produce osteoclasts increases with the purity of stem cells. Previous studies reported that macrophage colony-stimulating factor (M-CSF) activated the differentiation of HSCs into monocytes/macrophages; furthermore, mature cells of the monocyte/macrophage lineage could form osteoclasts, and immature monocytes and macrophages could also form osteoclasts when bone marrow stromal cells provided the appropriate microenvironment [ 65, 66].

In addition, bone and bone marrow contain three distinct macrophage populations, namely, osteoclasts and bone marrow macrophages, haematopoietic stem cell macrophages and osteal macrophages, which also suggests that osteoclasts and macrophages may have similar origins. It has been shown that cells from the monocyte/macrophage system, such as hematopoietic marrow cells, blood monocytes and peritoneal macrophages, can develop into bone-resorbing osteoclasts; therefore, the osteoclast population can be classified within these series of cells. In fact, osteoclasts and macrophages are two differentiation products of myeloid precursors that compete with each other [ 67, 68]. Osteoclasts are generated through a series of processes. The transformation of hematopoietic stem cells (HSCs) to monocytes/phagocytes initiates the differentiation of osteoclasts, followed by the proliferation and differentiation of osteoclast precursors and finally maturation into osteoblasts with bone resorption capacity.

## The Role of Subchondral Osteoclasts in OA

Subchondral bone is crucial for OA onset. Under normal physiological conditions, the osteochondral unit comprises subchondral bone and articular cartilage. Articular cartilage provides a smooth surface for movement, whereas subchondral bone provides stability and support. Together, they ensure the proper function and health of joints. A growing body of evidence suggests that abnormal remodeling of the subchondral bone in OA patients occurs before and, to some extent, accelerates articular cartilage degeneration. Additionally, the subchondral bone may be the primary source of pain in OA patients, making it essential to the pathogenesis of this disease [ 69, 70]. In the early stages of OA, hyperactivation of subchondral bone remodeling due to excessive bone resorption has been proposed as a major pathological hallmark of OA [71]. Bone remodeling is a highly coordinated process, and under normal conditions, osteoclast-mediated bone resorption and osteoblast-mediated bone formation are balanced to ensure the maintenance of bone homeostasis [72]. However, when OA occurs, the number of osteoclasts in the subchondral bone significantly increases, leading to enhanced bone resorption and alterations in the microstructure and microenvironment of the subchondral bone. Multiple signaling pathways and molecules are involved in the recruitment of subchondral osteoclasts. First, RANKL binds to the RANK receptor on the surface of osteoclasts and activates osteoclasts, which is a critical step that drives the migration of osteoclasts to bone tissue. Several chemokines and chemotactic proteins, such as CCL2 and CX3CL1, can be produced in subchondral bone and attract osteoclasts to these regions [ 73, 74]. During osteoclast migration, proteases such as collagenase are involved in the degradation of the bone matrix, providing a pathway for the movement of osteoclasts. Adhesion molecules on the cell surface, such as integrins, are also involved in the migration of osteoclasts through bone [75]. The periosteal microenvironment in the bone marrow also plays an important role in the recruitment of osteoclasts, providing a suitable environment for survival and differentiation. It affects angiogenesis and the innervation of subchondral bone, thereby accelerating articular cartilage damage and causing joint pain [ 76, 77]. Hence, subchondral osteoclasts have extraordinary significance in OA.

The role of subchondral osteoclasts in OA is mainly reflected in the structural destruction of subchondral bone and articular cartilage, angiogenesis, and joint pain. In the early stage of OA, the number and activity of subchondral osteoclasts increase abnormally, and the rate of bone resorption increases significantly. This disrupts the balance of bone remodeling, resulting in increased loss of subchondral bone, enlarged bone marrow cavities, and decreased bone mineral density. Excessive bone resorption leads to irregularities in the subchondral bone, which can cause the formation of bone cysts. In the subchondral bone, these cysts are liquid or semisolid cysts that can cause bone pain and discomfort. Moreover, overactive osteoclasts secrete proteases and degrading enzymes, such as MMPs, and capture enzymes, which degrade the cartilage matrix and lead to structural destruction of articular cartilage [ 78– 80].

**Figure 2:**
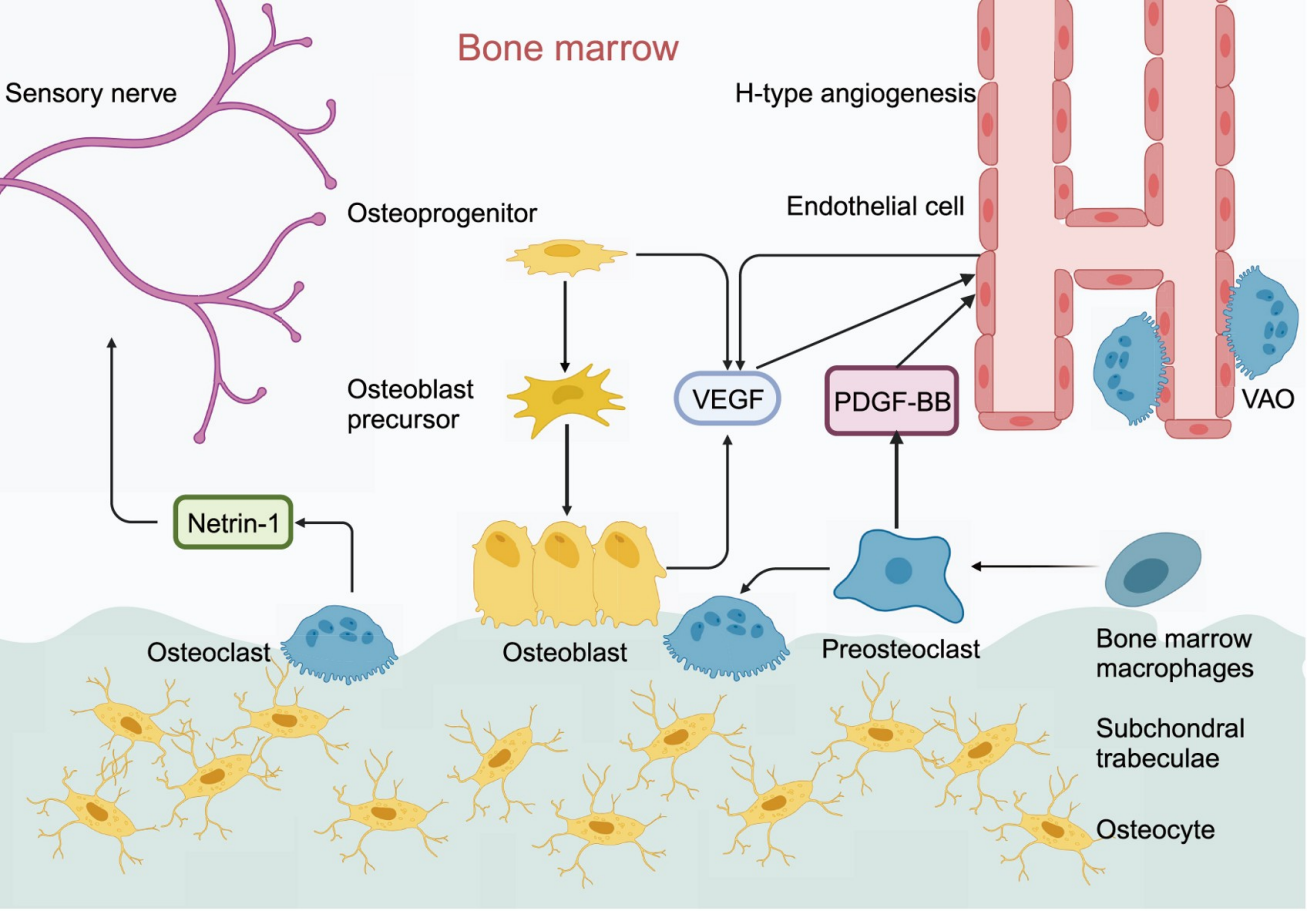
Figure 2 PDGF-BB secreted by subchondral osteoclast precursor cells promotes H-type angiogenesis in OA In the bone microenvironment, osteoblasts and endothelial cells secrete VEGF to promote H-type angiogenesis; at the same time, osteoclasts secrete Netrin-1 to act on sensory nerves, leading to joint pain in patients with OA.

CD31 ^hi^Emcn ^hi^, a specific vascular subtype, is an important feature of OA and was recently found to be closely associated with angiogenesis and osteogenesis [ 81, 82]. It is characterized by strong positive expression of platelet endothelial cell adhesion molecule (PECAM-1/CD31) and endothelial mucin (EMCN), which can exacerbate cartilage erosion in OA. Excessive secretion of PDGF-BB by osteoclast precursors induces the formation of the CD31 ^hi^Emcn ^hi^ vascular isoform ( Figure 2), and the number of CD31 ^hi^Emcn ^hi^ vessels increases significantly in OA. Moreover, vascular-associated osteoclasts (VAOs), a subtype of osteoclasts, can assist H-type blood vessels in eliminating cartilage [ 83– 86]. Moreover, excessive angiogenesis promotes osteogenesis. Despite increasing bone mass, it does not enhance bone strength. Instead, this leads to insufficient bone mineralization and destruction of the mechanical properties of the subchondral bone, which accelerates articular cartilage damage and exacerbates the vicious cycle of OA [87]. Sensory nerve fibers and nerve trunks are distributed within the vascular channels of articular cartilage and around the blood vessels of subchondral bone. Osteoclasts secrete Netrin-1, a protein that plays a key role in neural development and the function of the nervous system. This helps to establish neural connections by guiding the growth and migration of neuronal axons; thus, sensory innervation of the subchondral bone is related to the activity and number of osteoclasts during OA. Osteoclasts in subchondral bone introduce abnormal sensory innervation during OA, causing joint pain in OA patients [ 88, 89].

## Mechanism by Which Subchondral Osteoclasts Regulate OA

### Autophagy-related signaling pathways

Increasing evidence suggests that autophagy may play a pivotal role in regulating the proliferation, differentiation, and function of osteoclasts. Multiple signaling pathways, including the Beclin-1/Becn1, p62/sqstm1, mTOR, and HIF-1α pathways, play key roles in this process. Molecules such as CD147, G protein-coupled receptor kinase-interacting protein 1 (GIT1), IL-17A, and TRAF6 can regulate osteoclast autophagy through Beclin-1 [ 90, 91]. After CD147-mediated autophagy is activated, the levels of Beclin-1 and soluble RANKL increase, promoting osteoclastogenesis [92]. GIT1 promotes autophagy in osteoclasts by promoting the phosphorylation of the Beclin1 Thr119 site and disrupting the binding of Beclin1 to BCL2 [93]. IL-17A regulates RANKL-induced osteoclast formation by modulating Beclin-1-mediated autophagy [94]. TRAF6 mediates the ubiquitination of Beclin1 at Lys117 and promotes RANKL-stimulated osteoclast differentiation [95]. As a characteristic autophagy adaptor protein, p62/sqstm1 activates autophagy and is affected by LC3 accumulation and F-actin loop formation, which are involved in RANKL-induced osteoclast differentiation [96]. Additionally, mTOR regulates autophagy through the AMPK/mTOR/70-kDa ribosomal protein S6 kinase (P70S6K) signaling pathway, affecting osteoclast differentiation [97]. Among the protein signaling pathways through which HIF-1α regulates autophagy, the upregulation of BNIP3 is involved in hypoxia-induced autophagy activation [98]. Furthermore, HIF-1α mediates the involvement of miRNAs in autophagy regulation in osteoclasts [99].

### Noncoding RNAs (ncRNAs)

NcRNAs are important epigenetic regulators of osteoclast biological behavior. MiRNAs, circRNAs, and lncRNAs form a complex network that profoundly affects the biological activity of osteoclasts [100]. Among the miRNAs studied, miR-31 is one of the most upregulated miRNAs during osteoclastogenesis and regulates osteoclasts by affecting RhoA activity [101]. Moreover, miR-21, a new player in bone disease, promotes osteoclast formation and bone resorption through the PI3K/Akt signaling pathway [102]. Moreover, miR-34c promotes osteoclast survival by targeting leucine-rich repeat G-protein-coupled receptor 4 (lgr4) [103]. Mir-29b promotes osteoclast survival by inhibiting osteoclast apoptosis through the targeting of the proapoptotic factor Bcl-2 modifier (BMF) [104]. Mir-146-5p and mir-539 have been implicated in promoting osteoclast survival, bone resorption, and secretion, but their targets remain to be explored [ 105, 106]. A large number of circRNAs are upregulated during the early and late stages of osteoclastogenesis, suggesting that the expression profile of circRNAs is highly regulated during osteoclastogenesis. However, studies on the regulation of circRNAs by osteoclasts are rare. The available data indicate that circRNAs may function as miRNAs in the regulation of osteoclasts [107]. Exosomes are also involved in ncRNA communication. However, further studies are needed on the targeted regulation of subchondral osteoclasts by exosome-based ncRNAs.

### RANK/RANKL/OPG axis

The RANKL/RANK/OPG axis plays an essential regulatory role in osteoclast formation. This biological process begins with osteoblasts and stromal cells secreting RANKL, which interacts with osteoclast precursors and binds to RANK receptors. This interaction triggers the activation of a series of transcription factors, including NF-κB, activator protein 1, AKT, nuclear factor of activated T-cell cytoplasm 1 (NFATc1), and MAPK-related macromolecules such as ERK, JNK, and p38. Activation of these downstream factors initiates the transcription of genes related to osteoclast differentiation and bone resorption, including genes encoding anti-tartrate acid phosphatase (TRAP), cathepsin K (CTSK), the calcitonin receptor (CTR) and MMP-9. This process ultimately results in the formation of mature multinucleated osteoclasts [ 108– 113]. TNF receptor-associated factor 6 (TRAF6) is also an essential component of the RANKL-RANK signaling pathway, and recruitment of TRAF6 activates signaling pathways such as the NF-κB, MAPK, and PI3K/AKT pathways, thereby promoting osteoclast differentiation and maturation [ 114, 115]. In this process, the activation of NF-κB and the subsequent upregulation of transcription factors such as NFATc1 and c-Fos are critical steps in osteoclast differentiation and maturation [ 116, 117].

Moreover, OPG, a secreted protein and cytokine receptor protein produced by osteoblasts, plays a pivotal role in this process. It plays an important negative regulatory role in bone tissue. As a receptor antagonist, it can act as a decoy receptor to replace RANK and bind to RANKL, thereby inhibiting the formation of mature osteoclasts and consequently downregulating bone resorption. This mechanism is responsible for maintaining the homeostasis of bone tissue and ensuring that an appropriate ratio of bone resorption to osteogenesis is maintained. The expression of OPG is usually low but may be reduced in OA, leading to an increase in the combined effect of RANKL and RANK, which in turn increases the number and activity of osteoclasts, significantly increasing bone resorption and disrupting the balance of bone remodeling, thereby causing joint injury [ 118, 119]. Additionally, OPG can cause the breakdown of osteoclast pseudopods and protect the bone cortex through MAPK signaling and other pathways [120]. Overall, the RANKL/RANK/OPG axis is crucial for the development and function of osteoclasts. The ratio of OPG/RANKL determines the degree of bone resorption and the process of bone metabolism and is a crucial factor in bone and tissue metabolism.

### Oxidative stress

Oxidative stress-induced reactive oxygen species (ROS) play a large role in regulating the balance of the bone remodeling process. ROS include a variety of reactive molecules and free radicals, such as superoxide anions, hydrogen peroxide and hydroxyl radicals. These molecules are produced through the electron transport chain during aerobic respiration and can affect biological functions such as cell signaling and homeostasis [121]. Several recent studies have shown that under normal conditions, ROS are indispensable intracellular secondary messengers that perform numerous functions, including apoptosis, gene expression and activation of cellular signaling cascades, and play important roles in regulating cell proliferation, survival, metabolism, apoptosis, differentiation and migration [122]. However, ROS play dual roles. They are harmful when their levels increase due to aging or the onset of diseases such as OA. Excessive amounts of ROS can lead to bone destruction and even death of bone cells.

Recent studies have shown that oxidative stress and the consequent generation of ROS promote osteoclast differentiation. It has been demonstrated that RANKL stimulation increases ROS production in BMMs via the TRAF6/Rac1/nicotinamide adenine dinucleotide phosphate oxidase 1 (Nox1) signaling cascade, leading to enhanced osteoclast differentiation. In contrast, the antioxidant N-acetylcysteine (NAC) inhibited the response of BMMs to RANKL, which included ROS generation, MAPK pathway activation, and osteoclastogenesis. Similarly, in a glucose-induced diabetic rat model of osteoporosis, an increase in ROS production in osteoclasts was observed, followed by enhanced expression of MAPK, the NF- κB signaling pathway and NLRP3-related proteins, which promoted osteoclast differentiation and bone resorption [ 123, 124]. The production of ROS not only directly promotes osteoclast differentiation but also interacts with osteoblasts to regulate osteoclast formation and differentiation. The OPG/RANK/RANKL axis causes osteoblasts and osteoclasts to be inseparable [125]. High levels of H _2_O _2_-induced ROS in osteoblasts and BMSCs can stimulate RANKL mRNA and protein expression via the ERK and PKA-CREB pathways. The cocultivation of osteoblasts and osteoclast precursor cells demonstrated that the upregulation of RANKL expression induced by ethanol relied on the activation of intracellular ROS through NADPH oxidase activity in osteoblasts. Furthermore, the generated ROS actively facilitate the differentiation of osteoclasts [ 126, 127]. These findings imply that ROS play a pivotal role in enhancing RANKL secretion from osteoblasts, thereby modulating the differentiation of osteoclasts. In addition, the ROS/endoplasmic reticulum and ROS/TFEB pathways regulate osteoclast production and differentiation to a certain extent by affecting autophagy [ 128, 129].

### Inflammatory signaling pathway

Inflammatory cells are formed by immune cells, such as macrophages, T lymphocytes, B lymphocytes, and other leukocytes that infiltrate bone tissues and produce a variety of cytokines and chemokines, such as IL-1β, IL-6, TNF-α, nerve growth factor (NGF), and the anamnestic toxin C5a, all of which can regulate osteoclast activity to a certain degree, thus affecting OA. Accordingly, IL-1β can stimulate osteoclastogenesis by upregulating the expression of RANKL in osteoblasts or stromal cells, thereby significantly increasing the rate of bone resorption and disrupting the balance of bone metabolism, leading to the destruction of bone structure and accelerating the progression of OA [ 130, 131]. Simultaneously, IL-1β can affect osteoclasts through the NF-κB signaling pathway. When IL-1β binds to its cell surface receptor IL-1 receptor type I (IL-1RI), it causes receptor activation and the formation of a receptor complex consisting of IL-1RI, IL-1 receptor accessory protein (IL-1RAcP), and myeloid mediator protein 88 (MyD88). MyD88 activates IL-1RI and TRAFs through the IL-1 receptor-associated kinase (IRAK) to activate TRAF6. Activated TRAF6 stimulates TGF-β-activated kinase 1 (TAK1), which induces the expression of the kinase kappa B (IKK), leading to IκB protein degradation and NF-κB nuclear translocation, thereby regulating osteoclasts [132]. In addition, IL-1β activates the JAK-STAT and MAPK pathways to stimulate osteoclastogenesis [133]. Moreover, compared with IL-1β, IL-6 activates the JAK-STAT pathway and preferentially stimulates osteoclastogenesis. When osteoclasts are stimulated by IL-6 family cytokines, these factors bind to the gp130 receptor and activate gp130, activating JAK kinases, especially JAK1 and JAK2, which phosphorylate IL-6Rα. Activated STAT3 enters the nucleus and affects gene expression, thereby regulating osteoclast differentiation and activity [134]. In addition, IL-6 activates the NF-κB and MAPK signaling pathways, thereby regulating osteoclast activity and function. Like the RANKL/RANK system, TNF-α induces osteoclast differentiation but independently of this process. TNF-α recruits TRAFs to activate the transcription factors NF-κB, c-Fos, and NFATc1, which in turn induces osteoclast differentiation. However, TNF-α alone has a very limited role in inducing osteoclast formation, and several inhibitory proteins, including TRAF3, IRF8, and RBP-j, regulate this process [135]. Furthermore, NGF and the anamorphic toxin C5a can influence osteoclasts by activating the RANKL and MAPK signaling cascades, respectively [ 136– 138].

These cytokines and chemokines influence chondrocytes by interacting with osteoblasts and osteoclasts to regulate each other and maintain normal bone structure, thus indirectly affecting subchondral osteoclasts. The effects of the inflammatory factors IL-1β, IL-6, and TNF-α on chondrocytes can generally be summarized as catabolism, which induces further induction of inflammatory mediators as well as degradation of the cartilage extracellular matrix through the upregulation of a series of hydrolytic enzymes and moreover contributes to chondrocyte apoptosis [ 133, 139]. Chondrocytes can promote subchondral bone loss by regulating osteoclasts. Abnormal mechanical stress induces primary chondrocytes to produce IL-1b, which indirectly induces the differentiation and maturation of osteoclasts by increasing RANKL expression via osteoblasts. In the medial meniscus (DMM) instability-induced OA model, chondrocytes produce large amounts of TNF-α and IL-6. Moreover, TNF-α activated NF-κB and JNK in a Rankl-independent manner, which directly induced osteoclast differentiation and indirectly induced their production. In addition, senescent chondrocytes and hypertrophic chondrocytes produce proinflammatory mediators, catabolic enzymes, and chemokines, collectively referred to as senescence-associated secretory phenotypes (SASPs), which affect subchondral osteoclast lineage cells. In addition, osteoclast precursors invade the hypertrophic cartilage region and interact with chondrocytes to remodel the cartilage matrix and form ossification centers. Moreover, mature osteoclasts can regulate nearby chondrocytes, disrupting bone-cartilage connections and aggravating cartilage damage. TGF-β1 expression in osteoblasts was upregulated in a time-dependent and dose-dependent manner under mechanical stimulation. Chondrocyte apoptosis was aggravated when the cells were cocultured with osteoclasts. Intraperitoneal injection of a TGF-β1R inhibitor in OA rats effectively reduced chondrocyte apoptosis and cartilage degradation. TGF-β1 is transported from the subchondral bone to the cartilage layer by diffusion or blood circulation, which adversely affects chondrocytes. Chondrocytes affected by inflammatory factors can also indirectly affect osteoblast differentiation and maturation through ERK, NF-κB and other signaling pathways [ 33, 140].

### Other factors

**Figure 3:**
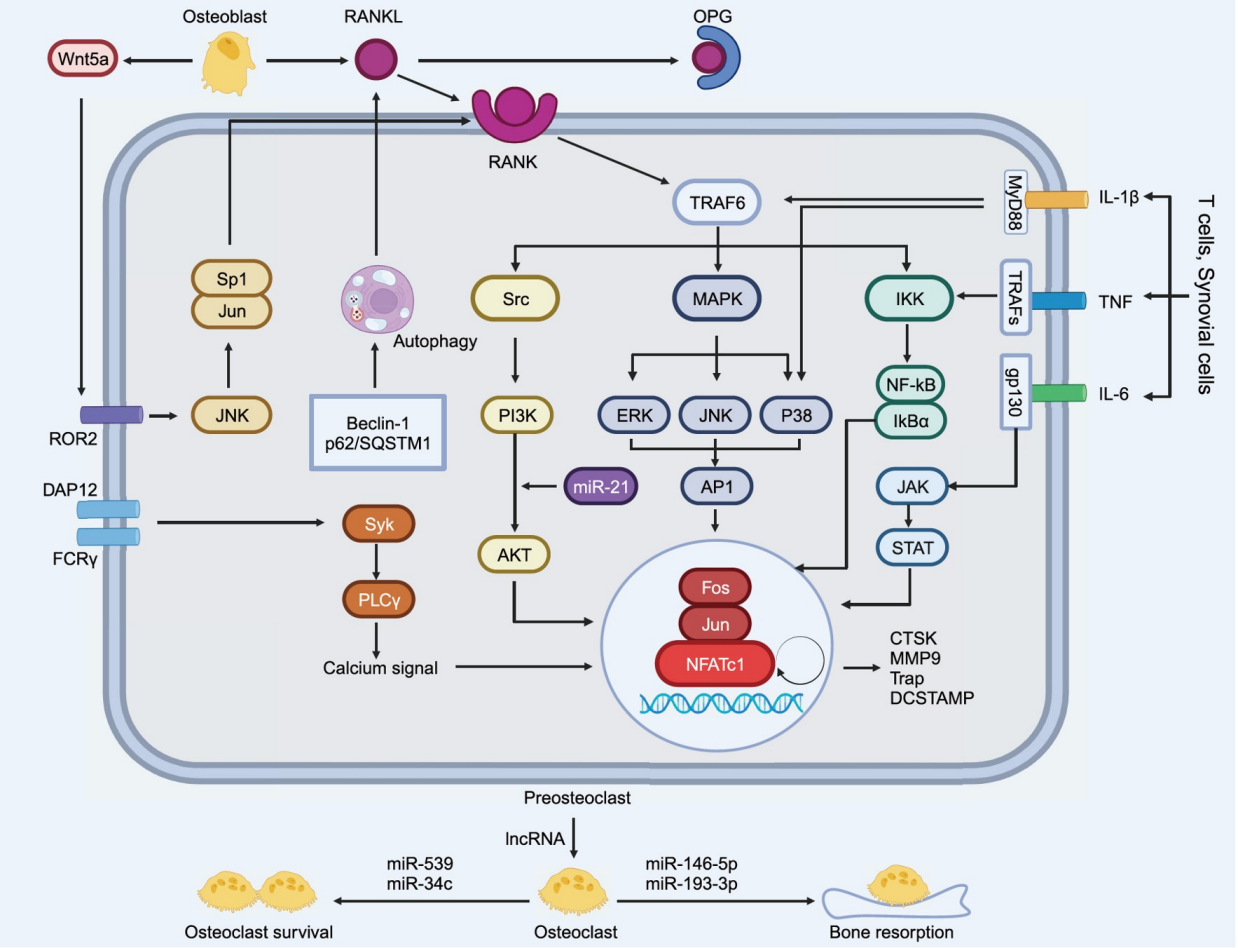
Figure 3 Mechanistic crosstalk of subchondral osteoclasts in OA The TRAF6 gene in the RANK/RANKL/OPG axis is the central factor that triggers the activation of a series of transcription factors, including NF-κB, AKT, MAPK, and NFATc1. Moreover, inflammatory factors and autophagy pathways are related to RANKL/RANK and interactively affect osteoclast differentiation; in addition, ncRNAs regulate osteoclast survival and bone resorption capacity.

The activity and function of osteoclasts in osteoarthritic subchondral bone are also affected by a variety of other factors, such as apoptosis [141], calmodulin [142], estrogen, thyroid proteins, Nrf2 [ 143, 144], RUNX2 [145], and other genes. These signaling pathways and factors work together to regulate osteoclast activity in OA ( Figure 3), leading to joint destruction and pain. An in-depth analysis of these regulatory mechanisms is important for understanding the pathophysiological processes of OA and developing relevant therapeutic approaches. By interfering with these signaling pathways, it is possible to alleviate symptoms and slow disease progression in patients with OA.

## Conclusions and Perspectives

Bone remodeling, which is essential for maintaining the mechanical capacity of the skeleton and coordinating the replacement of old and new bone, is a complex and subtle process mediated by all osteoblasts. Osteoclasts are the predominant cells involved in bone resorption and play an important role in maintaining bone remodeling. It has an indispensable function. OA is a chronic, degenerative joint disease that progresses irreversibly. Its main features are cartilage degeneration and abnormal remodeling of the subchondral bone. These lesions are inextricably linked to inflammatory processes. Different types of cells are involved in the inflammatory process. With the increasing understanding of the bone remodeling process and cellular activities in OA, subchondral osteoclasts appear to be the key to early pathological changes in OA and are expected to be a new target for the treatment of OA. In contrast to traditional NSAIDs or analgesics, treatments targeting subchondral osteoclasts focus on the underlying causes and pathophysiological mechanisms of OA, as opposed to pain and symptom relief, which is the main focus of traditional treatments. Targeted therapy can intervene in the pathophysiological processes that lead to subchondral osteolysis, such as inflammation, osteoporosis, and cartilage damage, thereby slowing disease progression and protecting joint structures, whereas traditional treatments can relieve only pain. Moreover, targeted therapies usually formulate treatment plans based on the disease characteristics and genotype of the patient and thus better meet the patient’s requirements. Moreover, targeted therapies can provide longer-lasting efficacy, whereas conventional therapies may require constant maintenance and lose effectiveness over time. Additionally, traditional therapies may cause gastrointestinal problems, liver and kidney damage, and other adverse effects. In contrast, targeted therapies typically have fewer systemic side effects due to their capacity to target arthritis-associated biomolecules with greater precision [ 146, 147].

Consequently, how can subchondral osteoclasts be targeted and regulated to achieve therapeutic effects? Currently, a large number of studies have shown that by inhibiting RANKL and activating AMPK, NF-κB. In addition, the generation of subchondral osteoclasts can be inhibited, bone resorption can be attenuated, and osteoclast-mediated abnormal remodeling of the subchondral bone can be reduced to alleviate OA. Drugs such as metformin, paroxetine, irisin, and some phenolactones significantly inhibit subchondral osteoclast differentiation and maturation ( Table 1). Additionally, from a mechanobiological point of view, mechanical cues and biochemical factors can modulate OA by affecting subchondral osteoclasts. There is growing evidence that appropriate mechanical loading reduces cartilage destruction, subchondral bone changes and secondary inflammation in OA joints. Some experiments have shown that early tibial axial mechanical loading may reduce the abnormal remodeling of subchondral bone and protect the cartilage from damage [148]; appropriate mechanical loading significantly reduces the level of IL-1β, as well as cox-2 and iNOS, and reduces the inflammatory state of OA joints through the NLRP3/caspase-1/IL-1β axis [149]; knee loading increases the expression of Wnt3a, and decreases the expression of NFATc1, RANKL, TNF-α, and Cathepsin K [150].

**Table:** **Table 1** List of drugs inhibiting subchondral osteoclast differentiation and maturation

Agent	Target	Signaling pathway	Function	Ref.
Metformin	BMMs	AMPK/NF-κB/ERK signaling	Inhibition osteoclast formation	[151]
Diterbutyl phthalate	BMMs/RAW264.7	ERK/c-fos/NFATc1 signaling	Inhibition of subchondral osteoclast formation and related angiogenesis and neurogenesis	[152]
Dihydroartemisinin	Osteoclast precursors	NF-κB/MAPK/NFATc1 signaling	Inhibition of osteoclast formation and bone resorption in the early stage of OA	[109]
Halofuginone	BMMs	Smad2/3-dependent TGF-β signaling	Inhibition of osteoclast bone resorption	[153]
Neratinib	ATDC5 cells/BMMs	MAPK/NF-κB signaling	Protect cartilage and inhibit osteoclast formation	[154]
Paroxetine	ATDC5 cells/BMMs	NF-κB signaling	Inhibition of pyrosis and osteoclast formation	[155]
Total lignans	BMMs	ERK/NFATc1 signaling	Inhibition of osteoclast differentiation	[156]
PP121	BMMs	RANKL/Src/MAPK/Akt signaling	Inhibition of osteoclast formation and bone resorption	[157]
Isorhamnetin	ATDC5 cells/BMMs	RANKL/ MAPK/ NF-κB signaling	Inhibit osteoclast formation and protect chondrocytes by regulating ROS homeostasis	[158]
Nirogacestat	BMMs	RANKL/NFATc1 signaling	Inhibition of osteoclast formation and bone resorption	[159]
USP13	BMMs	AKT/ NF-κB signaling	Inhibition of osteoclastogenesis and osteoclast related gene expression	[160]
Ruboxistaurin	BMMs	PKCδ/MAPKs signaling	Inhibition of osteoclast formation and absorption activity	[161]
Curcuminoid	RAW264.7	RANKL/OPG signaling	Reduce osteoclast activity and maintain osteoblast function	[162]
Irisin	ATDC5 cells/BMMs	RANKL/OPG/NF-κB signaling	Inhibition of osteoclast formation	[163]
FICZ	BMMs	RANKL signaling	Inhibit osteoclast differentiation and activity	[164]
Velutin	BMMs	RANKL/p38 signaling	Inhibits osteoclast formation and bone resorption	[165]
Parthenolide		NF-κB signaling	Inhibits osteoclast formation and survival	[166]
Lenalidomide	BMMs	RANKL/NF-κB signaling	Inhibits osteoclast formation and function	[167]
C-176	Osteoclast precursors	NF-κB/NFATc1 signaling	Inhibits osteoclast formation and activation	[168]
AZ-628	ATDC5 cells/BMMs	NF-κB/MAPK signaling	Inhibition of chondrocyte decomposition, osteoclast formation and bone resorption	[169]
Urolithin A	RAW264.7	RANKL/NF-κB signaling	Inhibition of osteoclast differentiation	[170]
HIF-2α inhibitor	BMMs	Akt/MAPK/NF-κB signaling	Inhibition of osteoclast differentiation	[171]
Diallyl disulfide	RAW264.7	RANKL/NF-κB/ NFATc1 signaling	Inhibition of osteoclast formation, fusion and bone resorption	[172]
Tyrosine kinase inhibitor	BMMs	RANKL/ NF-κB/ STAT3 signaling	Inhibition of osteoclast differentiation	[173]
Gypenoside	BMMs	RANKL/NF-κB/AKT/MAPK signaling	Inhibition of osteoclast formation	[174]

Additionally, biochemical factor levels are likewise not negligible in the regulation of subchondral osteoclasts. It has been shown that human OA articular cartilage stem cells suppress osteoclasts and improve subchondral bone remodeling in experimental knee OA partially by releasing TNFAIP3 [175]. It also inhibits overactive osteoclastogenesis and maintains the microarchitecture of subchondral bone by suppressing ROS production [176] and the expression of inflammatory mediators [177]. Additionally, lentiviral small hairpin RNA can knock down macrophage inflammatory protein 1 γ, thereby inhibiting osteoclast formation [178]. It can also inhibit osteoclast activity by inhibiting osteoclast-associated receptors (Oscar) [ 179, 180]. Moreover, exosomes derived from dental pulp stem cells (DPSCs) have been revealed to inhibit osteoclast activation in vivo by inhibiting TRPV4 activation and reducing cartilage degradation and synovial inflammation *in vivo* [181]. Targeted gene therapy can also regulate the activity and function of osteoclasts to achieve therapeutic effects. According to a previous article, ncRNAs, such as mir-21-5p, which targets Skp2 and can decrease osteoclast production, have great potential for treating OA [182]. Moreover, it has been shown that targeting and upregulating HMOX1 signaling can inhibit BMM-induced osteoclast activation, whereas selective knockdown of PDGF-BB in osteoclasts reduces subchondral bone angiogenesis and attenuates joint damage [183]. Moreover, numerous studies have shown that autophagy plays an indispensable role in regulating bone homeostasis and is expected to be a new target for regulating subchondral osteoclasts [ 184, 185]. Treatment of OA is a long-term process that focuses on improving disease management and quality of life. Future studies will continue to explore the molecular regulatory mechanisms of osteoclasts to identify new drug targets and pave the way for more effective treatment of osteoarthritis, and the development of individualized treatment strategies will provide more precise and effective treatments for OA patients, which will be a key focus of future research. In addition, the collaboration of multidisciplinary research teams will facilitate the integration of various studies on inflammation, bone resorption, and osteoclasts. In addition, the collaboration of interdisciplinary research teams will help to integrate multiple fields, such as inflammation, bone resorption, and osteoclast research, thereby providing new perspectives on the comprehensive treatment of OA.

In conclusion, this article reviews the latest progress on subchondral osteoclast differentiation in OA and targeted interventions for osteoclasts. Subchondral osteoclasts may play a central role in OA pathogenesis. We hope that this work will help us understand and develop new strategies for the targeted treatment of OA.
